# Detecting Noise-Induced Cochlear Synaptopathy by Auditory Brainstem Response in Tinnitus Patients With Normal Hearing Thresholds: A Meta-Analysis

**DOI:** 10.3389/fnins.2021.778197

**Published:** 2021-12-20

**Authors:** Feifan Chen, Fei Zhao, Nadeem Mahafza, Wei Lu

**Affiliations:** ^1^Centre for Speech and Language Therapy and Hearing Science, Cardiff School of Sport and Health Sciences, Cardiff Metropolitan University, Cardiff, United Kingdom; ^2^Department of Hearing and Speech Science, Guangzhou Xinhua College, Guangzhou, China; ^3^Department of Otolaryngology, The First Affiliated Hospital of Zhengzhou University, Zhengzhou, China

**Keywords:** tinnitus, cochlear synaptopathy, hidden hearing loss, central gain, auditory brainstem response, meta-analysis

## Abstract

Noise-induced cochlear synaptopathy (CS) is defined as a permanent loss of synapses in the auditory nerve pathway following noise exposure. Several studies using auditory brainstem response (ABR) have indicated the presence of CS and increased central gain in tinnitus patients with normal hearing thresholds (TNHT), but the results were inconsistent. This meta-analysis aimed to review the evidence of CS and its pathological changes in the central auditory system in TNHT. Published studies using ABR to study TNHT were reviewed. PubMed, EMBASE, and Scopus databases were selected to search for relevant literature. Studies (489) were retrieved, and 11 were included for meta-analysis. The results supported significantly reduced wave I amplitude in TNHT, whereas the alternations in wave V amplitude were inconsistent among the studies. Consistently increased V/I ratio indicated noise-induced central gain enhancement. The results indicated the evidence of noise-induced cochlear synaptopathy in tinnitus patients with normal hearing. However, inconsistent changes in wave V amplitude may be explained by that the failure of central gain that triggers the pathological neural changes in the central auditory system and/or that increased central gain may be necessary to generate tinnitus but not to maintain tinnitus.

## Introduction

Tinnitus is defined as a phantom sound without any corresponding external acoustic stimulus (Langguth et al., 2013). Long-term noise exposure, either occupational or recreational, is identified as the most common cause of tinnitus (Axelsson and Prasher, 2000). Tinnitus is often reported by patients with elevated hearing thresholds and, consequently, hyperactivity along the peripheral, and central auditory pathways after cochlear damage has been proposed as the primary cause (Jastreboff, 1990; Rauschecker et al., 2010; Roberts et al., 2010). It seems contradictory, therefore, that some 8–27.5% of tinnitus patients show a relatively normal performance in pure-tone audiometry (Sanchez et al., 2005; Zhao et al., 2010; Sheldrake et al., 2015). This suggests that normal hearing audiometry does not necessarily indicate normal cochlear function.

Recent research proposed that tinnitus with normal hearing thresholds may be explained by noise-induced cochlear synaptopathy (CS), which is a loss of synapses between the inner hair cells (IHCs) and auditory nerve (AN) fibers after excessive noise exposure (Kujawa and Liberman, 2009). Two types of AN fibers exist: high spontaneous discharge rate (SR) of fibers have low response thresholds, whereas low-SR fibers may be only activated by high threshold stimulation (Liberman, 1978). Notably, low-SR fibers are more vulnerable to noise exposure, by which the synaptic ribbons of IHCs could immediately and permanently be damaged [Furman et al., 2013; for review see Hickox et al. (2017)]. Since auditory brainstem response (ABR) wave I amplitudes represent the neural synchronization strength from spiral ganglion neurons (SGNs) into the auditory nerves, reduced suprathreshold wave I amplitudes could serve as a good proxy of loss or degeneration of low-SR fibers (Melcher and Kiang, 1996). Since most high-SR fibers remain intact, for hearing function in quiet, the threshold level still performs normal, which may explain normal audiograms in animal tinnitus models (Hickox et al., 2017).

Noise-induced tinnitus with normal hearing may result from increased central gain modulated by the homeostatic plasticity (Schaette and McAlpine, 2011). Homeostatic plasticity allows neurons to adjust their activity level within a dynamic range to respond to changes in synaptic inputs (Turrigiano, 1999). Thus, reduced activity of AN fibers may trigger increased central gain that enhances excitatory inputs and decreases the inhibitory inputs of downstream neurons to the ascending auditory pathway (Schaette and Kempter, 2006). This could be demonstrated by increased (or normal) ABR wave V amplitudes, which originate from the inferior colliculus (IC) at the level of brainstem (Melcher and Kiang, 1996). In addition, the ratio of wave V to wave I specifically indicates the magnitude of hyperactivity from the cochlear neurons (CN) to the IC (Schaette and McAlpine, 2011). Evidence obtained from animal studies showed increased spontaneous activity of the dorsal cochlear neurons (DCN; Middleton et al., 2011; Wu et al., 2016) and IC (Longenecker and Galazyuk, 2011) several days after noise exposure in different animal models with the sign of tinnitus, which supported the hypothesis of increased central gain to generate tinnitus. In tinnitus patients with normal hearing thresholds (TNHT), increased V/I ratios further implied the effect of central gain to generate tinnitus after deafferentation of AN fibers (Schaette and McAlpine, 2011; Gu et al., 2012; Nemati et al., 2014; Song et al., 2018; Valderrama et al., 2018).

Although the reduction of wave I amplitude in TNHT was detected by several studies (Schaette and McAlpine, 2011; Gu et al., 2012), some studies showed neither evidence of CS nor increased central gain (Guest et al., 2017; Shim et al., 2017), suggesting that ABR may not be sensitive to detect CS and/or central gain. Indeed, ABR could be influenced by some factors such as age (Grose et al., 2019), sex (McFadden and Champlin, 2000), hearing status of higher frequencies (Verhulst et al., 2016), or performance of distortion product otoacoustic emissions (DPOAE) (Bramhall et al., 2018).

On the other hand, it is also possible that low-SR fiber loss is not sufficient to generate tinnitus. Notably, tinnitus did not occur in animals where there was lower IHC ribbon loss (low-SR fiber) even with similarly reduced wave I amplitudes (Rüttiger et al., 2013). Knipper et al. (2013) hypothesized that tinnitus may result from a failure of induced central gain in the central auditory system. They suggested that it is the severe loss of high-SR fibers rather than low-SR fibers that mainly contributes to the generation of tinnitus (Knipper et al., 2020). High-SR fibers contribute to maintaining the auditory inhibitory network at central level (Singer et al., 2014). If a critical loss of high-SR fibers occurs, hyperactivity in the central auditory system may be explained by the reversal to excitation rather than disinhibition (Knipper et al., 2020). In this case, tinnitus is the result of increased neural noise rather than increased central gain, which is hypothesized to account for hyperacusis instead (Zeng, 2013). Recent studies using animal models have demonstrated a critical loss of IHC ribbons (to high- and low-SR fibers) related to reduced wave V amplitudes in animals with tinnitus-related behavior (Rüttiger et al., 2013; Singer et al., 2013). However, there is little consistent evidence in human study, despite one with tinnitus participants who had mild hearing loss (Hofmeier et al., 2018).

Although Milloy et al. (2017) reviewed the ABR findings on tinnitus patients with and without hearing loss, small numbers of studies for TNHT (*n* = 5) and missing ABR wave V data made it difficult to evidence CS and increased central gain in TNHT. There has been an increase in papers investigating CS by ABR in tinnitus patients with normal hearing since 2017. Thus, it is useful to reanalyze changes in ABR waveforms combined with new published papers. The primary aim of this study is to present a meta-analysis of ABR wave I and V amplitude to review the evidence of noise-induced CS and its possible effect on the central auditory system in tinnitus patients with normal hearing thresholds. This meta-analysis may also bring insights to two hypotheses of noise-induced tinnitus and corresponding neural effects in the central auditory system.

## Materials and Methods

### Search Strategy

Searches were conducted on September 5, 2021. PubMed, EMBASE, and SCOPUS databases were selected to search for relevant literature. Search terms were designed to identify all relevant papers: (tinnitus[Title]) AND (ABR^*^[Title/Abstract] OR auditory brainstem response^*^[Title/Abstract] OR brainstem response^*^[Title/Abstract] OR brainstem potential^*^[Title/Abstract] OR electrophysiology^*^[Title/Abstract]). The review followed the structure recommended by PRISMA to improve the quality and reporting of meta-analyses (Moher et al., 2009).

### Study Selection

Two authors (FC and NM) independently screened the title and abstract of identified papers. Since the research of noise-induced CS began to be well-conducted after the study by Kujawa and Liberman (2009), journal articles published after 2009 were added as an inclusion criterion. Only clinical human studies that utilized ABR as one of the main measurements were included. Literature reviews, case reports/series, meta-analyses, and animal studies were excluded. Participants (at least one group) in the included studies were required to be adults with normal audiometry and without a history of ear surgery, severe brain injury, tumors or ototoxic drug use. Table 1 summarizes the key components of the inclusion and exclusion criteria. Since the criteria of normal hearing varied across studies, we did not define the normal hearing audiometry but listed the criteria used by the included studies (Supplementary Table 1).

**Table 1:** Inclusion and exclusion criteria for searching.

	**Detailed items**
Inclusion criteria	Participants: Chronic tinnitus with normal hearing thresholds. Publication type: Peer-reviewed journals; published after 2009; in English. Outcome measure: Measured ABR wave I and V amplitudes, wave V/I and/or I/V ratio.
Exclusion criteria	Participants: pulsatile tinnitus; history of ear surgery, severe brain injury, tumors or ototoxic drug use; psychological disorders. Study design: animal studies, case reports/series, reviews, meta-analyses, conference articles, editorials. Study objective: studies investigating genetics, histology or treatment outcomes.

*ABR, auditory brainstem response*.

### Data Extraction

The two authors extracted the information and data independently. The second author (FZ) was involved when a discrepancy occurred. General characteristics of the studies were collated and listed in Supplementary Table 1, including participant characteristics (e.g., sample size, sex, and age), tinnitus characteristics (definition, pitch, and loudness matching), noise exposure history, hearing thresholds, and ABR results. ABR methodologies are summarized in Table 2 including the device model, transducer model, polarity of stimulus, type of stimulus, duration, sound level, stimulated rate, repetition, and filters.

**Table 2:** ABR methodology of the included studies.

**References**	**Device**	**Transducer**	**Stimulus type**	**Polarity**	**Duration**	**Sound level**	**Stimulated rate**	**Repetition**	**Filters**
Schaette and McAlpine (2011)	Medelec Synergy T-EP system	Telephonics TDH 49 headphones	clicks	N/A	50 μs	90, 100 dB SPL	11/s	90 dB: ≥8,000 100 dB: ≥6,000	100–1500
Gu et al. (2012)	Tucker-Davis Medusa	Sennheiser, HDA-200 headphones	clicks	Condensation	100 μs	30,50,70,80 dB nHL	11/s	30 dB: 15,840 50, 70, 80 dB: 7,920	5–5,000
Nemati et al. (2014)	ICS CHARTR	earphones	clicks	Alternating		90 dB SPL	11/s	2,000	N/A
Gilles et al. (2016)	Bio-Logic Auditory Evoked Potentials	N/A	clicks	Alternating	100 μs	80 dB nHL+ 55 dB nHL masking	31/s	2,000	100–3,000
Konadath and Manjula (2016)	Biologic Navigator Pro	N/A	clicks	Rarefaction	100 μs	70 dB nHL	11.1/s	1,500	30–3,000
Guest et al. (2017)	BioSemi ActiveTwo	EARtone 3A insert earphones	clicks	N/A	N/A	102 dB peSPL	14.1/s	7,040	30–1,500
Shim et al. (2017)	Navigator Pro	ER-3A insert earphones	clicks	N/A	N/A	90 dB nHL+30 dB nHL masking	13.3/s	1,500	100–3,000
Bramhall et al. (2018)	Intelligent Hearing Systems SmartEP	N/A	4 kHz tone burst	Alternating	2 ms	80, 90, 100, 110 dB peSPL	11.1/s	80, 90, 100 dB: 2,048 110 dB: 1,024	10–1,500
Hofmeier et al. (2018)	GSI Audera	Telephonics TDH 39p headphones	clicks	N/A	100 μs	25–75 dB SPL in 10 dB steps	11.1/s	2,000	150–3,000
Song et al. (2018)	Navigator Pro	N/A	clicks	N/A	N/A	90 dB	N/A	N/A	N/A
Valderrama et al. (2018)	SmartEP with Continuous Acquisition Module	ER-3A insert earphones	clicks	Rarefaction	113 μs	108.5 dB peSPL	39.1/s	12,500	200–2,000
Joo et al. (2020)	Navigator pro	N/A	clicks	N/A	N/A	90 dB	N/A	N/A	N/A

*N/A, not applicable*.

### Quality Assessment

A critical appraisal was conducted to determine the methodological quality of the included studies using the Newcastle–Ottawa Scale (NOS) (Stang, 2010). The NOS uses a star system to assess the quality of cohort studies based on three dimensions: selection, comparability, and outcomes. The results of individual studies were classified from one to nine stars with one star representing the highest quality for each item, except for a comparability item that could be awarded two stars. A final score of 0–3 was indicated as high risk of bias, 4–6 as medium risk of bias, and 7–9 as low risk of bias.

### Data Synthesis

Meta-analysis was conducted in Review Manager (RevMan, Version 5.4), The Cochrane Collaboration, 2020. The primary assessment investigated the amplitudes of ABR waves I and V and V/I ratio in normal hearing participants with or without tinnitus. Hedges's g was used, and a standardized mean difference (SMD) was calculated for the effect size with a 95% confidence interval (CI). A random effect model was chosen, and weighting of individual studies was calculated by combining the impact of the quality assessment and sample size. Similar to Cohen's *d*, 0.2, 0.5, and 0.8 of *Z* represents small, medium, and large effects, respectively. *I*^2^ is used to estimate the heterogeneity of individual studies contributing to the pooled estimate. The degree of heterogeneity was set at low (<40%), medium (40–60%), and high (>60%).

Sensitivity analysis was performed by excluding each study, in turn, to determine the influence of each individual study on overall estimates. Subgroup analysis was performed to determine the source of any heterogeneity. Various characteristics of participants were extracted as moderators, such as age, sex, and sound level of stimuli.

## Results

**Figure 1:**
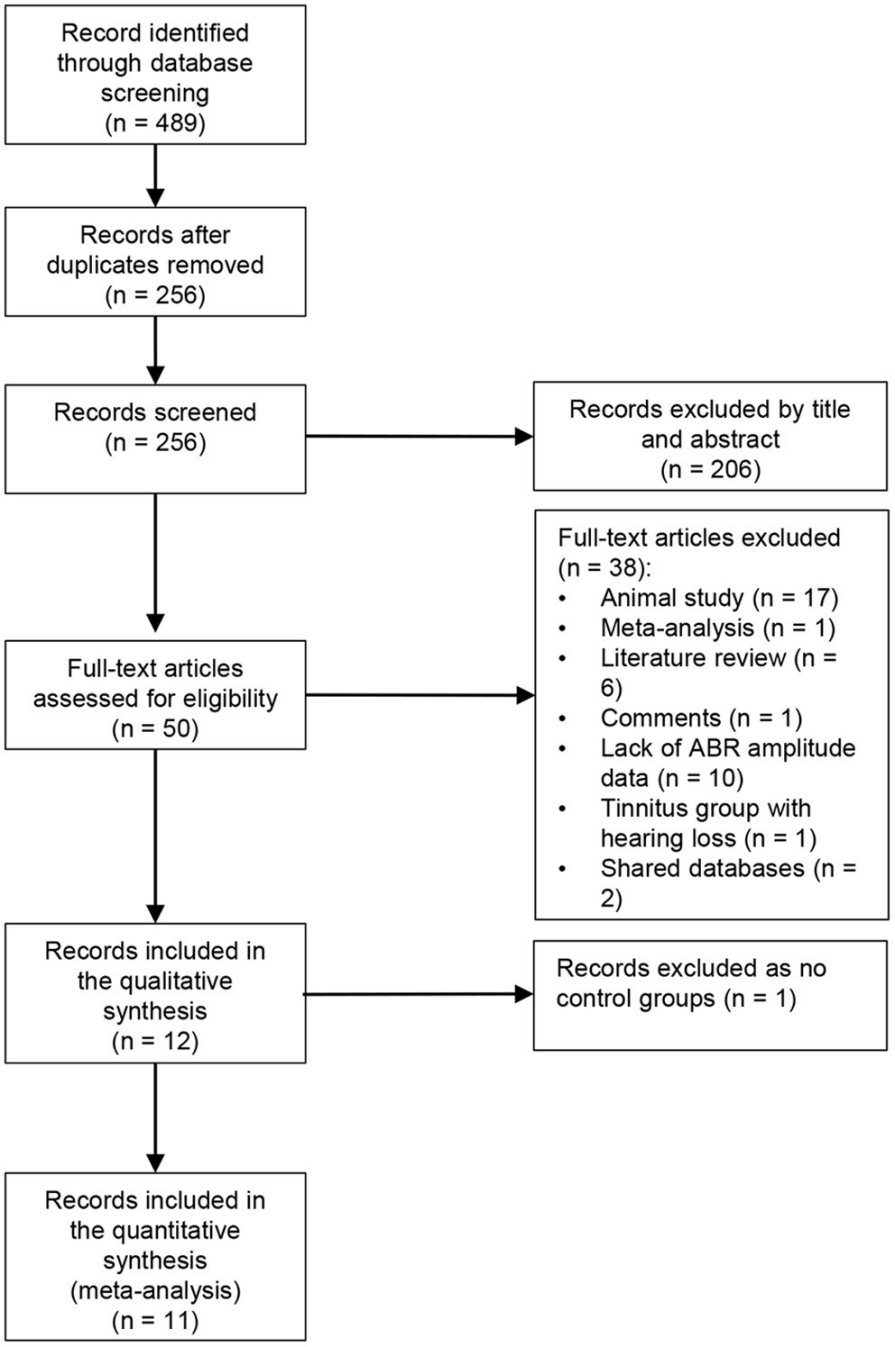
Flow diagram of study selection (following PRISMA).

A total of 489 publications were retrieved by the search terms. After removing duplicates, the title and abstract of 256 papers were screened. The full text of 50 papers were assessed for eligibility. Reasons for excluding papers are shown in Figure 1. It should be noted that although Hofmeier et al. (2018) recruited some participants with mild hearing loss ( ≤ 40-dB HL at a single frequency), the mean hearing thresholds in both groups from 0.125 to 8 kHz were within 20-dB HL. Finally, 12 studies were included for quality assessment and 11 for meta-analysis (Figure 1). Missing data or raw data were requested, and eight studies elicited a response.

### Demographic Characteristics of Included Studies

Supplementary Table 1 summarizes the demographic characteristics in the included studies. The sample size ranged from 33 to 128 (median: 51). The mean age of the tinnitus and control groups were 35.91 and 33.09, respectively, but the range varied widely. Gender distribution in nine studies were equal or nearly balanced. One study included only female participants (Schaette and McAlpine, 2011) and another only males (Gu et al., 2012). Most tinnitus participants in Bramhall et al. (2018) were males (male vs. female: 13 vs. 2), which could be explained by the military experience in the high-noise exposure group.

Noise exposure history was evaluated in six studies, whereas two studies excluded participants with a noise-related history. However, it should be noted that no structured interview or questionnaire for lifetime noise exposure was presented, which might create risk of bias (Nemati et al., 2014; Konadath and Manjula, 2016). The recruitment of noise-exposed participants in another study relied mainly on self-reporting (Gilles et al., 2016), which could also be biased. In contrast, three studies measured occupational and leisure noise exposure using an interview (Guest et al., 2017) or questionnaire (Bramhall et al., 2018; Valderrama et al., 2018). Notably, the structural interview by Guest et al. (2017) applied a different strategy to estimate noise exposure dose (>80 dBA). The sound level of individual events and activities were calculated with the sum providing total lifetime noise exposure [for details, see Guest et al. (2018)].

The inclusion criteria for tinnitus participants varied between studies. Six studies used the duration of tinnitus. The type of tinnitus was defined or collected in seven studies, and no participants with pulsatile tinnitus were recruited except the study by Valderrama et al. (2018). Since pulsatile tinnitus is usually triggered by the alteration in blood flow and different from noise-induced tinnitus (Hofmann et al., 2013), it may create a risk of bias. Four studies measured the psychoacoustic characteristics of the tinnitus, including localization (*n* = 3), pitch (*n* = 3), loudness (*n* = 4), minimum masking level, and residual inhibition (*n* = 1). The functional or emotional impact of tinnitus was evaluated by different questionnaires in six studies.

Gilles et al. (2016) suggested that recreational noise exposure was the most possible cause of tinnitus, though this was based on the self-report. Although tinnitus is observed frequently in people exposed to high-level noise, the pathophysiology of noise-induced tinnitus could be different from other types that are generated by different risk factors.

Hearing thresholds were measured in all studies. Notably, in the study of Hofmeier et al. (2018), some participants had no more than 40-dB HL in each frequency, which could make it hard to exclude the confounding effect of mild outer hair cell (OHC) loss from CS to ABR waveforms. By contrast, Gu et al. (2012) did not define the normal hearing threshold range of participants or report the average hearing thresholds in both groups. Hearing status at extended high frequencies, from 9 to 16 kHz, was evaluated in five studies, with three studies reporting no significant difference from the control group (Schaette and McAlpine, 2011; Gilles et al., 2016; Guest et al., 2017). Valderrama et al. (2018) defined no more than 40-dB HL from 8 to 12.5 kHz. In OAE results, only five studies measured DPOAE or TEOAE (Nemati et al., 2014; Gilles et al., 2016; Bramhall et al., 2018; Song et al., 2018; Valderrama et al., 2018). Three used OAE results as one of the inclusion criteria, with signal-to-noise ratio >3 or 6 dB from 1 to 4 kHz used most frequently. Lack of OHC status from OAE results may make it more difficult to interpret the reasons for changes in ABR amplitudes.

### Parameters Used for Auditory Brainstem Response Measurements

The details of ABR methodology used in each study are presented in Table 2. Eleven studies applied a click stimulus, whereas Bramhall et al. (2018) used a 4-kHz tone burst as the stimulus. Six studies reported the polarity of stimulus. Three applied condensation (Gu et al., 2012) or rarefaction (Konadath and Manjula, 2016; Valderrama et al., 2018), whereas three used alternating polarity (Nemati et al., 2014; Gilles et al., 2016; Bramhall et al., 2018). Notably, it is recommended that rarefaction should be used rather than condensation to produce enhanced amplitudes of ABR waveforms. Alternating polarity should be avoided to minimize artifacts (Hall, 2006). The polarity of the 4-kHz tone burst stimulus was reported as rarefaction by Bramhall et al. (2018), which is the same as recommended (Hall, 2006).

**Figure 2:**
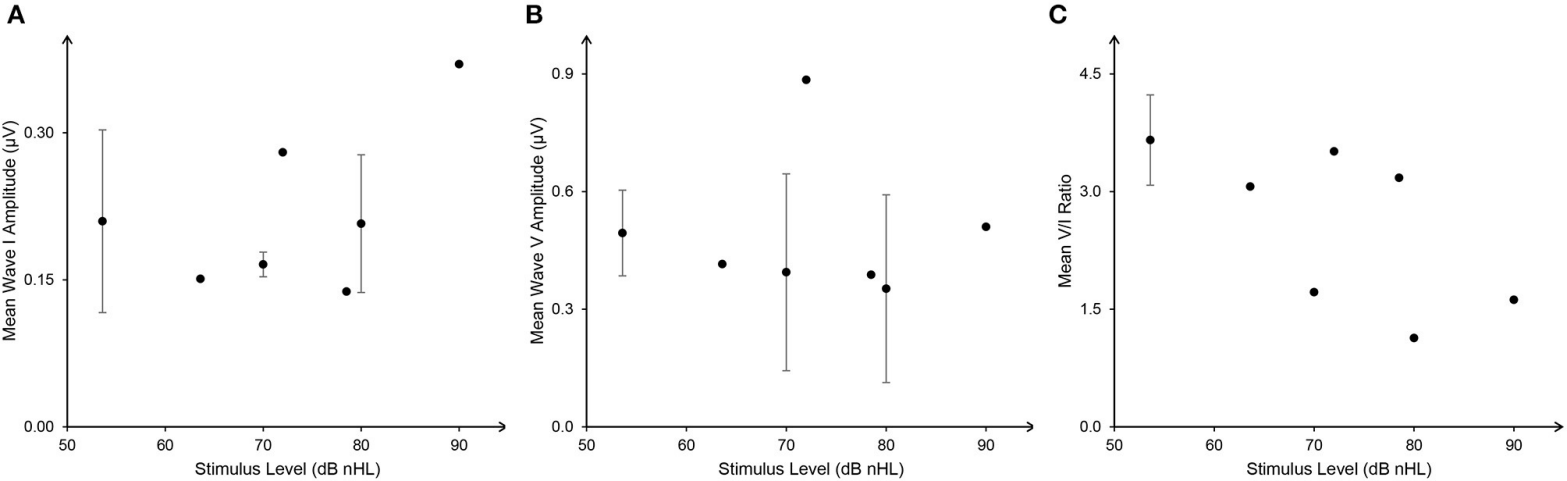
The weighted relationship between stimulus level [dB normal hearing level (nHL)] and auditory brainstem response (ABR) wave I **(A)**, V amplitude **(B)**, and V/I ratio **(C)** (mean ± SD). ABR data with dB sound pressure level (SPL) and dB peak-equivalent sound pressure level (peSPL) were converted into dB nHL with the formula: 0-dB nHL = 36.4 peak SPL = 29.9 peSLP (in a condition of 100 μs and 20/s).

Durations of clicks or tone burst were reported in seven studies. Four presented clicks of 100 μs, while Schaette and McAlpine (2011) used 50 μs, and Valderrama et al. (2018) set 113 μs. Bramhall et al. (2018) used a tone burst of 2 ms. Although most studies presented the stimuli at high intensity, the sound levels of the stimuli varied between studies with dB normal hearing level (nHL), dB sound pressure level (SPL), or dB peak-equivalent SPL (peSPL) being used (Table 2). In order to compare the ABR results, dB SPL and dB peSPL were converted into dB nHL using the formula 0-dB nHL = 36.4 peak SPL = 29.9 peSPL (in a condition of 100 μs and 20/s; Hall, 2006), as shown in Figure 2. Although the relationship between stimulus level and mean wave I and V amplitude were inconsistent, V/I ratios showed a negative tendency with increase in stimulus level. It should also be noted that even the ABR results at a single stimulus level varied widely across the studies, questioning the sensitivity of wave I amplitude to detect CS.

In addition, 10 studies reported stimulus rate. Eight studies used 11/s or similar. The clicks rates of the other studies were over 30/s (Gilles et al., 2016; Valderrama et al., 2018). ABR amplitude may decrease if the presentation rate increases over 31.1/s (Hall, 2006). Although 21/s was recommended by Bramhall et al. (2019) for investigating cochlear synaptopathy in a human ABR study, limited evidence from the included studies supports that recommendation.

Six studies applied between 1,500 and 2,000 sweeps, which according to Hall (2006) is sufficient to produce a confident SNR ratio for identifying wave V latency and amplitude. Thus, it seems effective to use 1,000 or 2,000 sweeps of clicks at high-level intensity (>90-dB SPL; Bramhall et al., 2019). In addition, filters were used in eight studies, with low- and high-pass filters included (Table 2). A low-pass filter is especially recommended to exclude the potential effect of OHC loss at higher frequencies (Bramhall et al., 2019). High-pass filters were highly variable, from 5 to 200 Hz, between studies. It should be noted that high pass over 100 Hz should be avoided (Hall, 2006). However, Gu et al. (2012) used a much lower-frequency high-pass filter (i.e., 5 Hz). The influence of using a lower-frequency high-pass filter may result in an increased amplitude of ABR waveforms, which could be contaminated by artifacts (Hall, 2006).

### Quality Assessment

The results of the quality assessment of the included studies are shown in Table 3. Two studies had high risk of bias (Konadath and Manjula, 2016; Joo et al., 2020), while six studies had medium risk of bias, and four studies had low risk of bias. Specifically, all but two studies provided details of tinnitus participant recruitment and defined inclusion and/or exclusion criteria. The participants reported in the included studies were typical tinnitus cases, though the tinnitus characteristics varied from individual studies. Six studies, however, did not recruit controls from the community, and the definition of controls was not given in several studies. As for comparability, based on the aim of the current study, the first and second impact factors were noise exposure history and OAE status. Three studies had two stars and another three received one star. In addition, noise exposure history of both the tinnitus and control groups were measured by three studies (Supplementary Table 1). Furthermore, although several studies reported missing data, the reason for those in the study of Valderrama et al. (2018) included technical problems, which did not meet the standard.

**Table 3:**
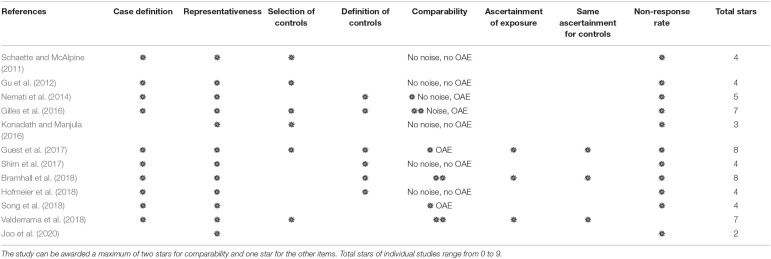
Quality assessment of the included studies by Newcastle-Ottawa-Scale (NOS) questionnaire.

*The study can be awarded a maximum of two stars for comparability and one star for the other items. Total stars of individual studies range from 0 to 9*.

### Meta-Analysis

**Figure 3:**
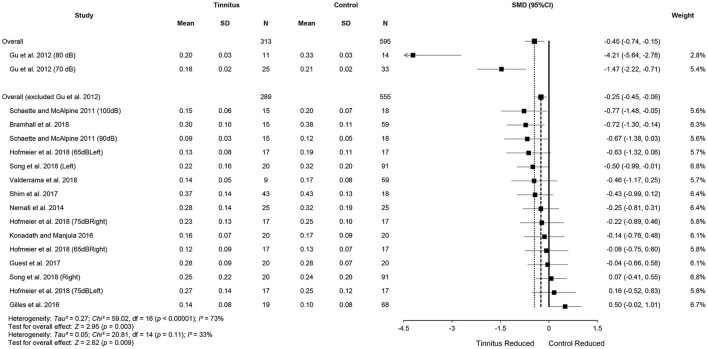
Forest plot of 11 studies for the difference of wave I amplitudes (95% CI) between tinnitus and control participants.

Data from the 11 included studies were extracted for meta-analysis of the ABR wave I amplitude. This included 313 tinnitus ears and 595 control ears (Figure 3). There was a significant difference in wave I amplitude between the tinnitus participants and controls (SMD = −0.45, 95% CI: −0.74, −0.15, *p* < 0.001), with lowered wave I amplitudes in the tinnitus participants. Although total SMD reduced to −0.25 (95% CI: −0.45, −0.06) after excluding two substudies with a large effect size [70 and 80 dB of Gu et al. (2012)], the significant difference of wave I amplitude between the two groups remained (*p* < 0.05). The result showed a large heterogeneity across the studies (*Chi*^2^ = 59.02, *p* < 0.001, *I*^2^ = 73%), and 33% heterogeneity still remained when the study by Gu et al. (2012) was removed. Several reasons might account for the reduction, such as potential loss of OHCs at the higher frequencies or the combined effect of age-related CS because of a much higher mean age (42 ± 6) of the participants (Supplementary Table 1). Condensation polarity may decrease ABR amplitude when compared with rarefaction polarity by producing an outward direction of basilar membrane movement that is opposite to that when afferent auditory nerves activate (Hall, 2006). It should be noted that Gu et al. (2012) used a much wider bandpass filter from 5 to 5,000 Hz. As a result, they showed a larger amplitude of wave I. In addition, the mean amplitude of controls at 80-dB nHL in the study of Gu et al. (2012) was much higher than two studies that used similar stimulus levels (Gilles et al., 2016; Konadath and Manjula, 2016) (Figure 3).

**Figure 4:**
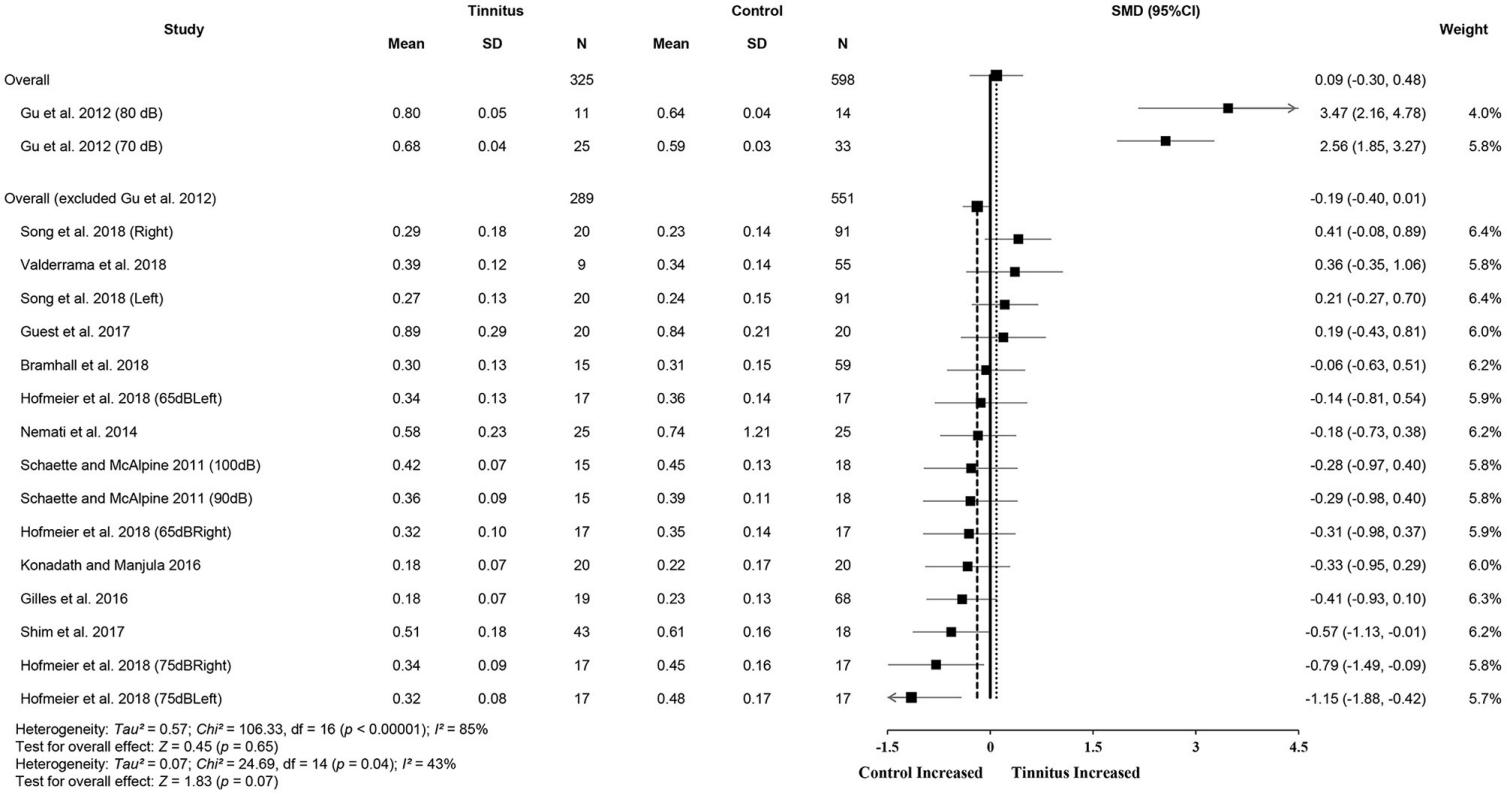
Forest plot of 11 studies for the difference of wave V amplitudes (95% CI) between tinnitus and control participants.

**Figure 5:**
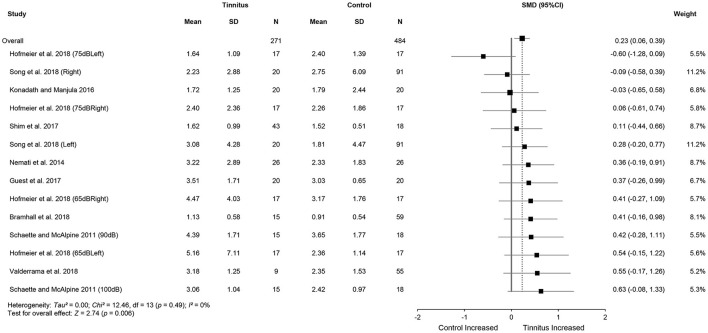
Forest plot of nine studies for the difference of wave V/I amplitude ratios (95% CI) between tinnitus and control participants.

Meta-analysis for ABR wave V amplitude included 325 tinnitus ears and 598 control ears from 11 studies (Figure 4). The results showed no significant difference in wave V amplitude between tinnitus participants and controls (SMD = 0.09, 95% CI: −0.30, 0.48, *p* = 0.65) and large heterogeneity (*Chi*^2^ = 106.33, *p* < 0.001, *I*^2^ = 85%). Heterogeneity decreased to 43% after removing the study of Gu et al. (2012). This result could be explained by the different measurement methods used in the study by Gu et al. (2012), i.e., wave V amplitude from prestimulus baseline to peak in comparison with the measures from peak to the following trough used in other studies. It is noteworthy that alteration in wave V amplitudes may not be consistent with the central gain hypothesis, given that eight studies showed reduced wave V amplitudes or a tendency for the reduction in tinnitus participants (Knipper et al., 2020). Data in nine studies (tinnitus ears 271 and control ears 484) showed significantly increased V/I amplitude ratios in tinnitus participants (SMD = 0.23, 95% CI: 0.06, 0.39, *p* < 0.05), which is consistent with the central gain hypothesis. The results indicated no heterogeneity across the included studies (*Chi*^2^ = 12.46, *p* = 0.49, *I*^2^ = 0%) (Figure 5).

Subgroup analysis was conducted to investigate the source of heterogeneity in wave I amplitudes. Sex, age, noise exposure history, and polarity were examined. Although there was no significant effect identified, sex may have had an influence (Table 4) as the overall reduction in the female tinnitus subgroup was larger than in the males (SMD = −0.53, 95% CI: −0.87, −0.19, *p* < 0.05) with no heterogeneity in the subgroups and no significant difference between the two subgroups (*Chi*^2^ = 2.26, *p* = 0.13).

**Table 4:** Subgroup analysis of the relationship (95% CI) between wave I amplitude and sex, age, noise exposure history and polarity.

**Subgroup**	**Sample size (** ***n*** **)**		**SMD (95%CI)**	***p-*value**
	**Tinnitus**	**Control**		
**Overall (sex only)**	183	223	−0.35 (−0.58, −0.13)	
Sex				0.13
Male	91	112	−0.19 (−0.48, 0.11)	
Female	92	111	−0.53 (−0.87, −0.19)	
**Overall (other subgroups)**	289	555	−0.25 (−0.45, −0.06)	
Age				0.55
<30	54	147	−0.08 (−0.79, 0.64)	
>30	235	408	−0.30 (−0.48, −0.13)	
Noise exposure history				0.81
No history	45	45	−0.2 (−0.61, 0.22)	
Investigated	63	206	−0.16 (−0.73, 0.4)	
Not investigate	181	304	−0.4 (−0.7, −0.11)	
Polarity				0.93
Alternating	59	152	−0.15 (−0.85, 0.56)	
Rarefaction	29	79	−0.28 (−0.74, 0.19)	
Not report	201	324	−0.29 (−0.49, −0.09)	

There was no significant difference between the young and older age groups (*Chi*^2^ = 0.35, *p* = 0.55). However, wave I amplitudes in the older subgroup were much lower than the controls and with little heterogeneity (*I*^2^ = 0%), which supports the hypothesis of age-related synaptic loss in AN fibers (Sergeyenko et al., 2013). There was no significant correlation between noise exposure history and wave I amplitude (*Chi*^2^ = 0.43, *p* = 0.81). While the results of the two subgroups (investigated and no history) were no different, this could be attributed to the limited sample size, and it would be unwise to neglect the effect of measurement of LNE on wave I amplitude reduction.

Notably, three studies with a younger subgroup were included in the investigated subgroup (Gilles et al., 2016; Guest et al., 2017; Bramhall et al., 2018), which may be able to explain the relatively large heterogeneity (*I*^2^ = 72%) within that subgroup (Supplementary Table 1). Polarity had no significant effect on wave I amplitude between the three subgroups (*p* = 0.86).

**Figure 6:**
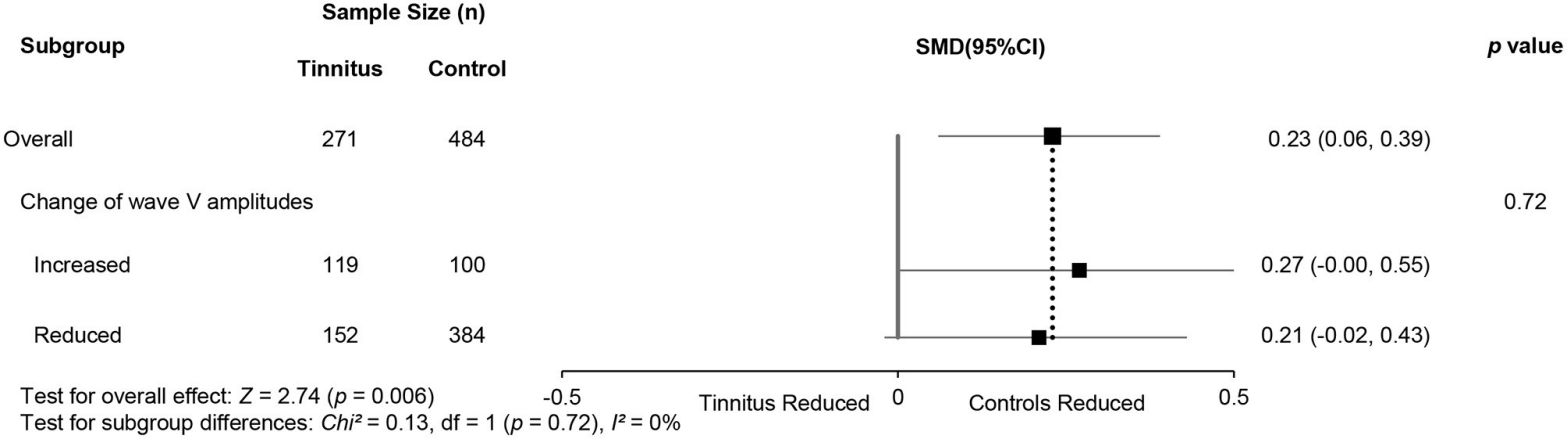
Subgroup analysis of the relationship (95% CI) between wave V/I ratios and changes in wave V amplitudes.

Subgroup analysis of wave V amplitudes produced no significant differences. This may indirectly suggest that two hypotheses for noise-induced tinnitus coexist (Table 5) and that there is a possibly combined, rather than contradictory, role of two types of CS in generating noise-induce tinnitus. Interestingly, there were also consistently increased V/I ratios in the reduced wave V amplitude subgroup (Figure 6), either indicating that the distinct regions contribute to increased central gain, or there is enhanced evoked activity that could be associated with hyperacusis. However, more evidence is needed to verify this speculation.

**Table 5:** Subgroup analysis of the relationship (95% CI) between wave V amplitude and sex, age, noise exposure history, and polarity.

**Subgroup**	**Sample size (** ***n*** **)**		**SMD (95%CI)**	***p*-value**
	**Tinnitus**	**Control**		
**Overall (sex only)**	183	219	−0.27 (−0.54, −0.00)	
Sex				0.69
Male	91	112	−0.21 (−0.62, 0.19)	
Female	92	107	−0.33 (−0.70, 0.05)	
**Overall (other subgroups)**	289	551	−0.19 (−0.40, 0.01)	
Age				0.67
<30	54	147	−0.13 (−0.47, 0.22)	
>30	235	404	−0.22 (−0.48, 0.03)	
Noise exposure history				0.55
No history	45	45	−0.24 (−0.66, 0.17)	
Investigated	63	202	−0.03 (−0.37, 0.30)	
Not investigate	181	304	−0.28 (−0.61, 0.04)	
Polarity				0.82
Alternating	59	152	−0.23 (−0.54, 0.08)	
Rarefaction	29	75	−0.01 (−0.68, 0.67)	
Not report	201	324	−0.23 (−0.53, 0.07)	

## Discussion

This review investigated whether the ABR changes in tinnitus patients with normal hearing are consistent across studies. The results show significantly reduced wave I amplitudes with low heterogeneity and increased wave V/I amplitude ratios. The changes in wave V amplitudes were inconsistent. No interaction was identified by subgroup analysis to explain the heterogeneity shown in reduced wave I amplitudes.

### Reduced Wave I Amplitude: Loss of Low-Spontaneous Discharge Rate Fibers or High-Spontaneous Discharge Rate Fibers

Synaptic ribbon, a presynaptic structure at active zones of the IHC synapse, tethers a large number of vesicles that enable a sustained high rate of transmission to AN fibers (Glowatzki and Fuchs, 2002). Transmitter release at these synapses enables precise temporal and intensity information to be passed to the auditory neurons for the accurate coding of time and intensity (Goutman and Glowatzki, 2007). The IHCs generate action potentials and transmit them to the central auditory system *via* AN fibers (Robles and Ruggero, 2001). Acoustic trauma has been linked to the loss of synapses between the IHC and the SGN in the terminals of type I AN fibers (Kujawa and Liberman, 2009; Rüttiger et al., 2013; Singer et al., 2013) and the disorganization of synaptic vesicles in the IHC cells (Bullen et al., 2019).

Notably, the roles of two types of AN fibers differ in auditory perception and processing. High-SR fibers determine the threshold of the auditory neural response at characteristic frequencies and are important for temporal resolution (Bourien et al., 2014). In contrast, low-SR fibers are important for hearing in noise during which high-SR fibers have become saturated (Costalupes et al., 1984; Costalupes, 1985). Moreover, high-SR fibers transmit envelop cues to the cochlear nucleus [for review, see Bharadwaj et al. (2014)], whereas low-SR fibers are involved in the coding of temporal fine structure and the temporal envelope, which account for speech intelligibility in noise (Lorenzi and Moore, 2007) or speech-on-speech masking release (Christiansen et al., 2013).

The reason why low-SR fibers are more vulnerable to acoustic damage may be because of fewer mitochondria present (Knipper et al., 2015). Deafferentation of low-SR fibers parallels the lower numbers of ribbons in the IHCs but with larger size, which in turn are correlated with the neural degeneration of SGNs (Kujawa and Liberman, 2009; Lin et al., 2011). Consequently, several animal studies suggested that loss of synapses in low-SR fibers would result in reduced spontaneous firing rates with no elevation of hearing thresholds [Lopez-Poveda and Barrios, 2013; for review, see Aedo and Aguilar (2020)]. Apart from ABR, the presence of CS in TNHT has been indicated by other measurements, such as damaged speech perception in noise (SpiN; Gilles et al., 2016) and increased SP/AP ratio in electrocochleography (EcochG; Kara et al., 2020), both of which have been linked with higher risk of cochlear synaptopathy in subjects (Liberman et al., 2016).

However, some animal studies have proposed that severe damage to high-SR, rather than low-SR fibers, induces noise-induced tinnitus (Rüttiger et al., 2013; Singer et al., 2013). The reduction of wave I amplitude was detected after noise exposure, but tinnitus-related behaviors linked with over 80% ribbon loss in high-frequency cochlear turns (Rüttiger et al., 2013). When there is extensive loss of ribbons in the synapses to high-SR fibers, response reliability of AN fibers degenerates along with reduced spontaneous and sound-evoked activity, reflected by prolonged latency and reduced amplitudes of wave I (Buran et al., 2010). Evidence showed that behavioral thresholds still were normal in mice, even though there was 95% loss of both low and high-SR AN fibers (Chambers et al., 2016). However, as higher degrees of ribbon loss cannot maintain the precision of the spike response of the AN fiber, hearing thresholds in the extended high frequencies also elevated without damage to the OHCs (Buran et al., 2010). This was consistent with the results in TNHT after noise overexposure (Sulaiman et al., 2014; Kumar and Deepashree, 2016; You et al., 2020). To support that, envelope following responses (EFR) in quiet were measured in the tinnitus group with normal audiometry, suggesting the damage to both high-SR and low-SR fibers, though the etiology of tinnitus in this study remained unclear (Paul et al., 2017).

Regardless of the types of AN fiber loss, so far, the presence of CS in tinnitus still remains inconsistent among studies from different measurements [for review see Bramhall et al. (2019)]. However, a possible role of synaptopathy in the generation of tinnitus in humans cannot be excluded. Noise exposure showed a close interplay with aging to CS in animals (Fernandez et al., 2015; Möhrle et al., 2016), which is accordant with the higher risk of tinnitus in older people (Al-Swiahb and Park, 2016). Noise-related effect could exaggerate the age-related synaptopathy, though such an effect was expected to be “acute” (Fernandez et al., 2015). In addition, the age-related neural degeneration between SGN and IHC was observed in temporal bones in humans (Wu et al., 2019). Therefore, younger individuals are likely more resistant to noise trauma than older participants, and as a result, the presence of CS in TNHT may be combined results of noise exposure and aging.

### Changed Wave V Amplitude: Central Gain or Failure of Central Gain

The current review found large heterogeneity in wave V amplitudes with increased or decreased amplitudes in tinnitus patients. Enhanced or normal wave V amplitude with decreased wave I amplitude is consistent with the consequences of central gain following deafferentation of low-SR fibers (Schaette and McAlpine, 2011). Yet decreased amplitudes supported the “failure of central gain to restore amplitudes completely,” which is hypothesized resulting from the severe loss of high-SR fibers (Knipper et al., 2020).

Several animal studies have suggested that loss of low-SR fibers reduced spontaneous and sound-evoked activities with a concomitant reduction of excitatory drive to the ascending auditory pathway (Puel et al., 1998; Wang and Green, 2011). However, homeostatic adaptation plays an important role in maintaining central gain by increasing excitation and decreasing inhibition to restore the reduced neural inputs, which could be reflected in normal or increased wave V amplitudes (Schaette and Kempter, 2006). Central gain is believed to originate between the CN and IC, where there are both excitatory and inhibitory interneurons that can interact to maintain homeostasis (Schaette and McAlpine, 2011; Auerbach et al., 2014; Sedley, 2019). Spontaneous neural activity in the central auditory system was enhanced after excessive noise exposure within a few hours or weeks and could persist in the absence of inputs (for review see Eggermont, 2017), including the DCN (Kaltenbach and Afman, 2000; Dehmel et al., 2012; Koehler and Shore, 2013), IC (Bauer et al., 2008; Mulders et al., 2010; Longenecker and Galazyuk, 2011; Manzoor et al., 2012), and the auditory cortex (AC; Sun et al., 2008; Basura et al., 2015; Eggermont, 2015; Vanneste and De Ridder, 2016). Consequently, tinnitus might be generated as a result of hyperactivity. In other words, tinnitus could be a side effect of homeostatic adaptation causing increasing spontaneous activity in the central auditory system [Schaette and Kempter, 2006, 2009; for review see Noreña (2011)].

However, the magnitude of central gain may depend on the degree of cochlear damage after noise exposure. While moderate damage produces central enhancement to compensate for reduced neural activity, severe damage to IHCs may fail to increase the spontaneous activity in the central auditory system (Schaette and Kempter, 2006). This is supported by reduced central ABR wave V amplitudes in both animal (Rüttiger et al., 2013; Singer et al., 2013; Möhrle et al., 2019) and human studies (Hofmeier et al., 2018). Loss of high-SR fibers could trigger the impairment of an inhibitory network. The development and maintenance of the fast-spiking parvalbumin-positive (PV^+^) inhibitory interneurons to cortical pyramidal neurons depend on the development of high-SR fibers after hearing onset (Chumak et al., 2016). When high-SR fibers are severely damaged, the inhibitory network could be reversed into hyperexcitation rather than disinhibition, resulting in the increased spontaneous activity at the central auditory system [for review see Knipper et al. (2020)]. In addition, the PV+ interneurons actively participate in bottom–up feedforward and top–down feedback inhibition to improve sound resolution through frequency-dependent contrast amplification (Knipper et al., 2020), which is consistent with the frequency-related characteristics of residual inhibition in numerous tinnitus patients (Roberts et al., 2008). Different findings from tinnitus research may support this hypothesis. A few complaints of tinnitus from people with congenital hearing loss may point out the importance of mature high-SR fibers for the pathophysiology of tinnitus (Eggermont and Kral, 2016; Lee et al., 2017). Another evidence is that diminished high-SR fibers limited the ability to properly attenuate irrelevant stimuli over relevant information, which were reported by some tinnitus patients (Delano et al., 2007; Wittekindt et al., 2014).

As for increased wave V amplitudes in some studies, Knipper et al. (2020) regarded them as the confounding effect of hyperacusis, considering that it is also believed to result from enhanced central gain. There is a high prevalence but lack of measurement in tinnitus patients (Schecklmann et al., 2014). One neuroimaging study identified increased activation in the IC and medial geniculate body (MGB) in patients with hyperacusis but cortical activation in tinnitus and hyperacusis (Gu et al., 2010). This parallels the hypothesis of Zeng (2013) that tinnitus is the result of increased loudness by enhanced central neural noise, but hyperacusis is the result of steeper loudness growth by central gain enhancement. Möhrle et al. (2019) instead proposed that central compensation may reflect a healthy status in homeostatic adaptation processes and their ability to stabilize the discharge rate of the central auditory system, which also contradicts the hypothesis of Schaette and McAlpine (2011).

Different results of central gain in the included studies may also indicate the contribution of central gain in distinct auditory structures either from cortical or subcortical levels, though the characteristics of tinnitus may cover the hyperacusis and render it undetectable. Notably, since only a few included studies measured hyperacusis in the tinnitus group, consistently increased V/I ratios may indicate the contribution of hyperactivity triggered by either or both types of deafferentation to the generation of tinnitus or hyperacusis. In particular, central enhancement for low-level stimuli, which is mainly due to high-SR fiber loss, may provide the neural basis of tinnitus, whereas neural gain for high-level sound triggered by low-SR fiber loss may account for the generation of hyperacusis (Salvi et al., 2016). On the other hand, inconsistent wave V amplitudes may result from different subtypes of tinnitus. Although loss of high-SR fibers could explain relevant hearing impairment and prolonged latency of wave V in tinnitus patients (Hofmeier et al., 2018), the function of OHCs was not assessed. Altered ABR wave V are potentially accounted for by OHC loss around the tinnitus frequency. According to the model proposed by Schaette and Kempter (2012), the cochlear damage that reduces sound-evoked input to the central auditory system could trigger homeostatic plasticity and cause tinnitus as a side effect.

### A Possible Explanation of Tinnitus With Normal Hearing Thresholds

Although numerous animal and human studies investigated how cochlear synaptopathy may generate tinnitus, a few studies considered seemingly contradictory findings of synaptopathic effect and the change in central gain in the time course of tinnitus. Animal results showed that noise exposure can result in a wide range of ribbon loss in the IHCs (30–80%), which could be from sole low-SR fiber loss to combined fiber loss (Kujawa and Liberman, 2009; Lin et al., 2011; Furman et al., 2013; Rüttiger et al., 2013; Singer et al., 2013). Wave I amplitude could be intact after mere low-SR fiber loss (Bourien et al., 2014) and start to decrease in any point of the following damage range (Knipper et al., 2015). A previous study found that monkeys might have a much smaller degree of synaptopathy than mice when their hearing thresholds are normal, suggesting that primates could be more vulnerable to the damage to hair cells (Valero et al., 2017). On the other hand, inconsistent changes of wave V amplitude (Figure 4) imply that the neural effect of central gain at lower level could disappear in long-term tinnitus (all included studies recruited chronic tinnitus). Thus, it is possible that increased central gain by low-SR fiber loss may provide the necessary neural basis for tinnitus with normal hearing, including the increased spontaneous and synchronized activity at the central auditory system. When the deafferentation aggravates (up to high-SR fibers), such loss reverses the inhibitory circuit into excitatory status, modulates top–down regulation, and eventually makes tinnitus persistent.

Such hypothesis could be partially supported by the findings that alternations of noise-induced central gain may vary at different levels and during different times [for review, see Auerbach et al. (2014)]. Amplitude from the IC reduced 1-h post-noise exposure and gradually increased to normal after a week, while the evoked responses from the AC amplified immediately after noise and showed similar amplitudes as previously 1 week later (Syka et al., 1994). However, this theory cannot be used directly into the tinnitus model and need to be further verified, considering that the source of noise exposure is much more complicated in the environment.

### Implications for Future Study

The present systematic review with meta-analysis attempts to gather all relevant studies and, thus, clarified whether and how the cochlear synaptopathy is involved in the mechanism of tinnitus in patients with normal hearing. The results derived from meta-analysis implied that two types of AN fiber loss may characterize different stages in the tinnitus generation and progress. It may lead to a new direction for future studies in noise-induced tinnitus.

Since cochlear synaptopathy is not able to be directly measured in the human study, it is very important to unify the definition of normal hearing thresholds in the future study, at least adopting a common one. The WHO updates the definition of normal hearing audiogram to that less than 20 dB in average of thresholds at 500, 1,000, 2,000, and 4,000 Hz (World Health Organization, 2021). Notably, since the hearing damage at high frequencies is very frequent after noise exposure, including the thresholds at 3,000, 6,000, and 8,000 Hz is highly recommended (Bramhall et al., 2019). Extended high frequencies from 12 to 16 kHz should be assessed as well. In addition, the characteristics of hyperacusis should be measured to avoid the confounding effect, since it is also believed to be triggered by increased central gain (Auerbach et al., 2014).

The latency of ABR wave I and wave V is recommended to better reveal the degree of degeneration in the AN fibers. It may also help to distinguish the high-SR fiber loss from low-SR fiber loss, considering the prolonged wave I latency when high-SR fibers were damaged (Buran et al., 2010). A future study is encouraged to recruit acute noise-induced tinnitus patients and compare with chronic tinnitus patients to find whether the synaptopathic effect on the central auditory system is different in the time course of tinnitus. A longitudinal study is highly recommended to track the possible role of CS as well as central gain in the maintenance of tinnitus. Moreover, future studies that include ABR and EEG or fMRI are expected to better investigate any cortical changes caused by CS in either the auditory or non-auditory regions.

## Conclusion

This review highlighted a significant reduction in wave I amplitude in tinnitus patients with normal hearing thresholds. Two possible hypotheses were discussed: increased central gain triggered by low-SR fiber loss or failure of central gain caused by high-SR fiber loss. However, neither of them could solely explain the inconsistency of wave V amplitude change. Consistently increased V/I ratio may indicate the contribution to central gain in different regions, which plays an important role in the generation of tinnitus and/or hyperacusis. Further study is recommended to investigate the subcortical and cortical changes along the auditory pathway caused by noise-induced cochlear synaptopathy, which helps to reveal the roles of two mechanisms in the generation and maintenance of tinnitus.

## Data Availability Statement

The original contributions presented in the study are included in the article/Supplementary Material, further inquiries can be directed to the corresponding author/s.

## Author Contributions

FC and FZ designed the study. FC and NM performed the literature search and data extraction. FC wrote the paper. FZ and WL provided critical revision of the manuscript. All authors contributed to the article and approved the submitted version.

## Conflict of Interest

The authors declare that the research was conducted in the absence of any commercial or financial relationships that could be construed as a potential conflict of interest.

## Publisher's Note

All claims expressed in this article are solely those of the authors and do not necessarily represent those of their affiliated organizations, or those of the publisher, the editors and the reviewers. Any product that may be evaluated in this article, or claim that may be made by its manufacturer, is not guaranteed or endorsed by the publisher.
