# Supplementary material for: Detecting Noise-Induced Cochlear Synaptopathy by Auditory Brainstem Response in Tinnitus Patients With Normal Hearing Thresholds: A Meta-Analysis
Source: Front Neurosci. 2021 Dec 20;15:778197. doi: 10.3389/fnins.2021.778197 (PMC8721093; doi:10.3389/fnins.2021.778197)
Supplement: Supplementary file 1 [file Table_1.docx]

**Supplemental TABLE 1 |** The general information of included studies and results of ABR amplitudes in tinnitus participants.

| **Study** | **Subjects** | **Age (yr) and Sex** | **Noise Exposure History** | **Tinnitus Characteristics** | **Hearing Thresholds** | **OAE Results** | **Wave I Amplitude** | **Wave V Amplitude** | **V/I Amplitude Ratio** |
| --- | --- | --- | --- | --- | --- | --- | --- | --- | --- |
| Schaette and McAlpine, 2011 | T: 15  C: 18 | 36.3 ± 2.6  33.2 ± 1.9  Females | N/A | > 6 months, Non-pulsatile, Modified tinnitus spectrum procedure (pitch and loudness) | 0.125–8 kHz  ≤20 dB HL,  12, 16 kHz:  no significant difference with controls | N/A | Reduced at 90 and 100 dB SPL** | Did not differ | Increased at 90 and 100 dB SPL |
| Gu et al. 2012 | T: 15  C: 21 | 42 ± 6  43 ± 7  Males | N/A | Duration, Localization, Type, Pitch, Loudness, MML, TRQ, RI | 0.125-16 kHz  But no reported results | N/A | Reduced at 80 dB nHL *** | Increased at 30 and 80 dB nHL * | Increased at 70* and 80 dB nHL *** |
| Nemati et al. 2014 | T: 25  C: 25 | 34.4 ± 12.2  33.8+11.5  M:F = 9:16  M:F = N/A | Subjects had no noise exposure history | > 3 months,  Non-pulsatile, Bilateral | 0.25-8 kHz  ≤25 dB HL | TEOAE using click stimuli at 80 dB SPL: SNR 6 dB at least three points from 1-4 kHz | Did not differ | Did not differ | Increased at 90 dB SPL** |
| Gilles et al. 2016 | T: 19  C: 68 | < 30  M:F = 11:8  M:F = 12:56 | Attended parties, concerts, festivals per week but did not attend 2 days before testing | > 3 months, Non-pulsatile,  Noise-induced (self-report), Duration,  Localization, Type, VAS (loudness), TQ | 0.125-16 kHz  ≤25 dB HL,  9-16 kHz: no significant difference from controls | TEOAE using 80 µs click stimuli at 80 dB SPL: SNR > 3 dB from 1-4 kHz;  DPOAE: SNR ≥ 6 dB from 1-4 kHz. | Did not differ | Did not differ | No report |
| Konadath and Manjula, 2016 | T: 20  C: 20 | 33.15 ± 9.80  20.50 ± 1.79  M:F = 10:10  M:F = 10:10 | Subjects had no noise exposure history | Continuous  THI > 38 | 0.25-8 kHz  ≤20 dB HL | N/A | Did not differ | Did not differ | Did not differ |
| Guest et al. 2017 | T: 20  C: 20 | 25.7 ± 1.3  25.3 ± 1.3  M:F = 10:10  M:F = 10:10 | History of LNE (> 80 dBA) (interview),  units of noise exposure | > 4 months, Constant,  Non-pulsatile, TFI | 0.125–8 kHz  ≤20 dB HL,  10, 14 kHz:  no significant difference from controls | N/A | Did not differ | Did not differ | Did not differ |
| Shim et al. 2017 | T: 43  C: 18 | 33.60 ± 13.42  28.61 ± 9.67  M:F = 19:24  M:F = 8:10 | N/A | > 6 months, Unilateral,  Not objective or somatic,  Duration | 0.25-8 kHz  ≤20 dB HL | N/A | Did not differ | Did not differ | Did not differ |
| Bramhall et al. 2018 | T: 15  C: 59 | 26.3 ± 2.1  26.7 ± 4.5  M:F = 13:2  M:F = 22:37 | LENS-Q | Constant, A questionnaire included a question about the perception of tinnitus | 0.25-8 kHz  ≤20 dB HL | DPOAE from 1-8 kHz, above the 90th percentile at all tested frequencies and below the 95th percentile at no more than one tested frequency were included | Reduced at 90, 100 and 110 dB pe SPL | Did not differ | Increased at 90, 100 and 110 dB pe SPL |
| Hofmeier et al. 2018 | T: 17  C: 17 | 36.53 ± 13.01  33.18 ± 13.49  M:F = 11:6  M:F = 11:6 | N/A | G-H-S tinnitus questionnaire: severity, localization, emotional distress,  cognitive distress, self-experienced intrusiveness, auditory perceptual difficulty,  Pitch, Loudness | 0.125-10 kHz  < 40 dB HL | N/A | Did not differ | Reduced at 75 dB SPL ** | N/A |
| Song et al. 2018 | T: 20  C: 91 | 37 ± 12.6  43 ± 11.3  M:F = 10:10  M:F = 40:51 | N/A | Non-pulsatile,  Duration, Localization, THI | 0.25-8 kHz  ≤20 dB HL | DPOAE from 1-12 kHz | Reduced only at 90 dB in the left side** | Did not differ | Did not differ |
| Valderrama et al. 2018 | T: 11  C: 63 | 42.18 ± 7.15  43.57 ± 6.83  M:F = 6:5  M:F = 32:31 | History of LNE (survey): sound level, duration, working hour, hearing protection; type of high-noise leisure activities, and use of hearing protection | Constant, Types | 0.25-6 kHz  ≤20 dB HL,  8,9,10,11.25,12.5 kHz ≤40 dB HL | DPOAE amplitude from 1-12 kHz | Did not differ | Did not differ | Increased at 108.5 dB pe SPL** |
| Joo et al. 2020 | T:128 | 47.9 ± 16.4  M:F = 5:15 (Acute),  34:49 (Subacute), 30:44 (Chronic) | N/A | Acute: < 1month,  Subacute: 1-6 month,  Chronic: > 6 month,  Duration | 0.5-8 kHz  ≤20 dB HL | N/A | Did not differ | Reduced at 90 dB in the subacute group** | Did not differ |

*Noted:* * represents *p* < 0.1, ** represents *p* < 0.05, *** represents *p* < 0.001, T = tinnitus, C = controls, MML = minimum masking level, TRQ = Tinnitus Reaction Questionnaire, RI = residual inhibition, M = males, F = females, SNR = signal to noise ratio, VAS = Visual Analogue Scale, TQ = Tinnitus Questionnaire, THI = Tinnitus Handicap Index, LNE = lifetime noise exposure, TFI = Tinnitus Function Index, LENS-Q = The Lifetime Exposure of Noise and Solvents Questionnaire, G-H-S = Goebel-Hiller-Score.
